# Ibrexafungerp: A Novel Oral Triterpenoid Antifungal in Development for the Treatment of *Candida auris* Infections

**DOI:** 10.3390/antibiotics9090539

**Published:** 2020-08-25

**Authors:** Mahmoud Ghannoum, Maiken Cavling Arendrup, Vishnu P. Chaturvedi, Shawn R. Lockhart, Thomas S. McCormick, Sudha Chaturvedi, Elizabeth L. Berkow, Deven Juneja, Bansidhar Tarai, Nkechi Azie, David Angulo, Thomas J. Walsh

**Affiliations:** 1 Department of Dermatology, Case Western Reserve University, Cleveland, OH 44106, USA; Mahmoud.Ghannoum@UHhospitals.org (M.G.); tsm4@case.edu (T.S.M.); 2 Unit of Mycology, Statens Serum Institut, DK-2300 Copenhagen, Denmark; maca@ssi.dk; 3 Department of Clinical Microbiology, Rigshospitalet, DK-2100 Copenhagen, Denmark; 4 Department of Clinical Medicine, University of Copenhagen, DK-2200 Copenhagen, Denmark; 5 New York State Department of Health Wadsworth Center, Albany, NY 12201, USA; vishnu.chaturvedi@health.ny.gov (V.P.C.); sudha.chaturvedi@health.ny.gov (S.C.); 6 Centers for Disease Control and Prevention, Atlanta, GA 30333, USA; gyi2@cdc.gov (S.R.L.); kuu4@cdc.gov (E.L.B.); 7 Max Super Specialty Hospital, 110017 New Delhi, India; devenjuneja@gmail.com (D.J.); Bansidhar.Tarai@maxhealthcare.com (B.T.); 8 Scynexis Inc., 1 Evertrust Plaza, 13th Floor, Jersey City, NJ 07302, USA; nkechi.azie@scynexis.com; 9 Transplantation-Oncology Infectious Diseases Program, Weill Cornell Medicine of Cornell University, New York, NY 10065, USA; thw2003@med.cornell.edu

**Keywords:** antifungal, ibrexafungerp, *Candida* *auris*, resistance

## Abstract

*Candida auris* is an emerging multidrug-resistant fungal pathogen reported worldwide. Infections due to *C. auris* are usually nosocomial and associated with high rates of fluconazole resistance and mortality. Echinocandins are utilized as the first-line treatment. However, echinocandins are only available intravenously and are associated with increasingly higher rates of resistance by *C. auris*. Thus, a need exists for novel treatments that demonstrate potent activity against *C. auris*. Ibrexafungerp is a first-in-class triterpenoid antifungal agent. Similar to echinocandins, ibrexafungerp inhibits (1→3)-β-D-glucan synthase, a key component of the fungal cell wall, resulting in fungicidal activity against *Candida* spp. Ibrexafungerp demonstrates broad in vitro activity against various *Candida* spp. including *C. auris* and *C. auris* isolates with *fks* mutations. Minimum inhibitory concentration (MIC_50_ and MIC_90_) values in >400 *C. auris* isolates were 0.5 μg/mL and 1.0 μg/mL, respectively. Clinical results were reported for two patients with invasive candidiasis or candidemia due to *C. auris* treated during the CARES (Candidiasis Caused by Candida Auris) trial, an ongoing open-label study. These patients experienced a complete response after treatment with ibrexafungerp. Thus, ibrexafungerp represents a promising new antifungal agent for treating *C. auris* infections.

## 1. Introduction

*Candida auris* is an emerging fungal pathogen reported on all continents except Antarctica, in at least 39 countries worldwide [1], as well as in 20 states of the United States [2,3,4,5]. Five distinct clades of *C. auris* were identified with well-defined geographic distributions (South America, Africa, South Asia, East Asia, and West Asia), as well as antifungal resistance patterns and mechanisms that are both distinct and unique [1,6,7,8]. Infections due to *C. auris* are most often nosocomial, with easy transmission from patient-to-environment and environment-to-patient [6,9]. Patients heavily colonized with *C. auris* on the skin or mucosal surfaces can contaminate their surroundings, thereby contributing to transmission of *C. auris* in healthcare facilities. An additional challenge with *C. auris* is that the organism is exceedingly difficult to eradicate from the environment because of resistance to some standard disinfectants [6,9,10].

Timely and accurate diagnosis of invasive candidiasis are important for early initiation of antifungal therapy, while species identification is critical to ensure implementation of infection control measures [1,6,10,11]. Acceptable standard diagnostic methods for *C. auris* identification include matrix-assisted laser desorption ionization–time of flight (MALDI-TOF) and VITEK2™ with the appropriate updated databases and DNA sequencing [7,12].

Infections caused by *C. auris* are associated with severe illness, most often in hospitalized patients. Risk factors for *C. auris* infection include prior exposure to antibiotics or antifungal agents, diabetes mellitus, abdominal or vascular surgery, central venous or urinary catheters, chronic kidney disease, chemotherapy, blood transfusions, immunosuppression, and intensive care unit admission [6,7,10,11]. Invasive infections due to *C. auris* are associated with high mortality rates (30–78%), and multidrug resistance may play a role in mortality [6,7,10,13].

An outbreak of infections due to *C. auris* was identified in New York healthcare facilities with high rates of mortality [13]. Since 2016, more than 1000 *C. auris* isolates were tested at the New York State Department of Health where rates of resistance were >99% with fluconazole, approximately 60% with amphotericin B, and >80% with voriconazole [14,15]. The in vitro efficacy of antifungal drug combinations was evaluated against these resistant *C. auris* isolates, where combinations of flucytosine with echinocandins or amphotericin B were most active [14].

The epidemiology of *C. auris* was examined from isolates obtained from 54 patients collected from six countries outside the US [16]. Patient information was available for 41 (76%) isolates. At the time of *C. auris* detection, 41% of patients had diabetes mellitus, 51% had undergone recent surgery, 73% had a central venous catheter, and 41% were receiving systemic antifungal therapy. From hospital admission to confirmed infection with *C. auris* the median time was 19 days, 61% of patients had bloodstream infection, and 59% of patients died. Although breakpoints are not established for *C. auris* against any antifungal drugs, tentative breakpoints were established by the CDC (Centers for Disease Control) based on minimum inhibitory concentration (MIC) distribution, molecular mechanisms of resistance, and PK/PD (Pharmacokinetic/Pharmacodynamic) values in a mouse model of infection. Based on these preliminary breakpoints, the authors determined that 93% of the isolates were resistant to fluconazole, 35% to amphotericin B, and 7% to echinocandins; 22 (41%) isolates were resistant to at least two antifungal classes, including two (4%) that were resistant to fluconazole, voriconazole, echinocandins, and amphotericin B.

As suggested in the IDSA (Infectious Diseases Society of America) guidelines for the treatment of *Candida* infections and given the high frequency of resistance to fluconazole and polyenes, echinocandins are typically utilized as the first-line treatment for patients with *C. auris* infections. Although resistance is clone and clade specific, recent reports indicate that global resistance of *C. auris* to fluconazole may approach 90%; this is increasing with other antifungal agents, including echinocandins [7,9,10,11,17]. Antifungal resistance with *C. auris* is acquired rather than intrinsic, and the primary mechanisms of resistance were characterized for echinocandins and azoles [8]. Multidrug-resistant and pan-resistant isolates of *C. auris* were also identified from clinical isolates [15,16,18]. Thus, a need exists for novel antifungal agents that demonstrate high levels of activity against *C. auris* and address these treatment gaps. There is also need for effective infection control practices as well as treatment strategies that minimize the risk of nosocomial transmission associated with persistent *C. auris* colonization to control its spread.

## 2. Ibrexafungerp

### 2.1. Mechanism of Action

**Figure 1:**
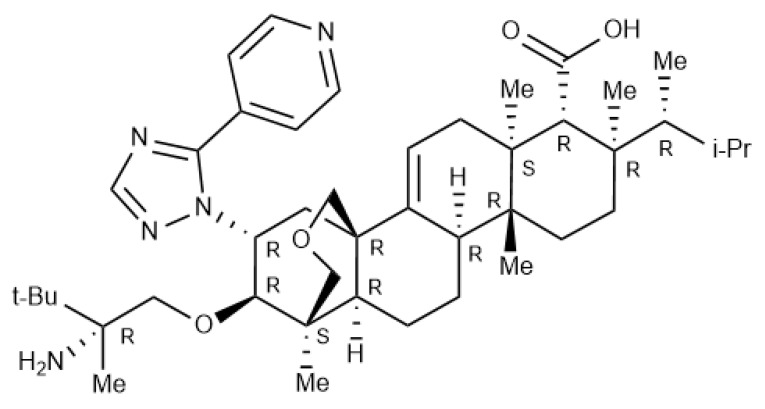
Structure of ibrexafungerp.

Ibrexafungerp (formerly SCY-078) is the first compound of the enfumafungin-derived triterpenoid class of (1→3)-β-D-glucan synthase inhibitors (GSIs) (Figure 1). Glucan synthase inhibitors were first introduced for the treatment of invasive *Candida* infections in 2001, with caspofungin the first echinocandin to be approved [19]. This mechanism of action, i.e., blockade of the biosynthesis of ß-(1,3)-D-glucan in the fungal cell wall, was associated with potent and broad-spectrum antifungal activity and clinical efficacy for the treatment of fungal infections. Two additional echinocandins were later introduced, micafungin and anidulafungin. However, echinocandins lack clinically meaningful oral bioavailability, triggering the search for new molecules that shared the glucan synthase inhibition mechanism of action with echinocandins and could also be administered orally. Natural screening efforts led to the identification of enfumafungin derivatives as candidates, and subsequent synthetic modifications to these molecules resulted in increased oral bioavailability, potency, and stability, thereby leading to the discovery of ibrexafungerp.

**Figure 2:**
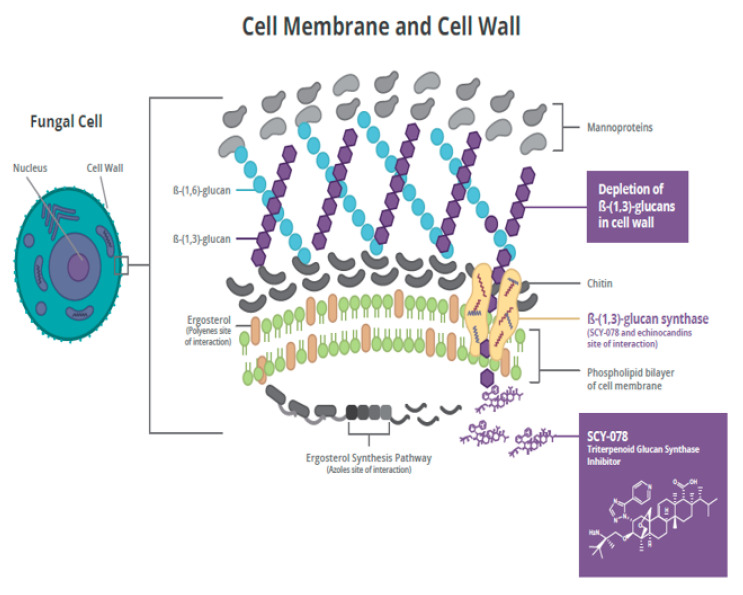
Mechanism of action for ibrexafungerp.

Ibrexafungerp is being developed as the first oral and IV GSI (Intravenous glucan synthase inhibitor) for the treatment and prevention of fungal infections, including serious and life-threatening infections due to *Candida* spp., *Aspergillus* spp., and *Pneumocystis jirovecii*, with the potential to provide the therapeutic advantages of both IV and oral formulations [20]. Ibrexafungerp causes a decrease in (1→3)-β-D-glucan polymers and a weakening of the fungal cell wall [21]. Ibrexafungerp is structurally distinct from echinocandins and interacts differently with the target enzyme (Figure 2) [22]. Although the binding site on (1→3)-β-D-glucan synthase for ibrexafungerp partially overlaps with a binding site for echinocandins, it appears to be nonidentical, resulting in a lower rate of resistance to ibrexafungerp [22]. In in vitro studies, ibrexafungerp activity against wild-type and echinocandin-resistant strains of *Candida* spp. in the presence of *fks* mutations was minimally affected [23]. Thus, ibrexafungerp has limited potential for cross-resistance with echinocandins.

### 2.2. In Vitro and In Vivo Activity

Ibrexafungerp demonstrates broad in vitro activity against a range of *Aspergillus* spp. isolates and *Candida* isolates, including *C. glabrata* and *C. auris,* which exhibit *fks1* and *fks2* point mutations associated with resistance to echinocandin antifungals [23,24,25,26,27,28,29,30,31]. Among *Candida* species with reduced fluconazole susceptibility, including *C. glabrata, C. krusei, C. tropicalis*, and *C. parapsilosis*, MIC_50_ ranges with ibrexafungerp were 0.125–1 μg/mL, 0.5–1 μg/mL, <0.03–1 μg/mL, and 0.25–1 μg/mL, respectively. Additionally, as reported by Zhu [32] using isolates obtained from New York patients, the in vitro activity against *C. auris* of ibrexafungerp (ranging from 0.05 to 0.5 μg/mL) was superior to that of fluconazole (ranging from 2 to >256 μg/mL), and comparable or superior to that of echinocandins (ranging from 0.015 to 16 μg/mL). This observation was confirmed by other studies using global strains [28,33,34]. Ibrexafungerp showed a wild-type MIC distribution against ~80% of echinocandin-resistant *Candida* spp. isolates tested, suggesting that *fks* mutations have less of an effect on the in vitro activity of ibrexafungerp [23,35].

Ibrexafungerp demonstrates potent fungicidal activity against *Candida* spp. [31,36,37,38]. In a time-kill study, ibrexafungerp demonstrated a ≥3-log reduction in colony forming units (CFUs)/mL) at 24 h. Caspofungin demonstrated fungicidal activity similar to ibrexafungerp, but fluconazole and voriconazole were fungistatic [31]. Ibrexafungerp was shown to have activity against biofilms from different *Candida* species [28]. Consistent with clinical trials in treatment of vulvovaginal candidiasis, ibrexafungerp showed potent in vitro activity in the lower pH environment of vulvovaginitis [39].

### 2.3. Pharmacokinetics

After oral administration in dog, mouse, and rat, ibrexafungerp is absorbed rapidly from the gastrointestinal (GI) tract, with bioavailability of approximately 35–50% [40]. In animals, ibrexafungerp is widely distributed in tissues with a steady-state volume of distribution (V_dss_) of >5 L/kg [41], which is several-fold greater than fluconazole and echinocandins [38]. Higher V_dss_ results in higher tissue-to-plasma concentration ratios could be beneficial for specific fungal infections, such as those located in the skin, liver, spleen, mucosa, bone, and lung, to mention some. Ibrexafungerp undergoes extensive metabolism by cytochrome P450 3A4 isoenzymes in the liver, and <2% of a dose is recovered unchanged in urine [42]. After single oral doses of 10 mg to 1600 mg in healthy subjects, peak ibrexafungerp plasma concentrations were reached after four to six hours, with a mean terminal half-life of approximately 20 to 30 h [43]. Mean area under the concentration-time curve (AUC_0-∞_) and peak concentration (C_max_) were dose-proportional across this dosage range. The proarrhythmic potential of ibrexafungerp was evaluated in animal models and healthy volunteers using cardiodynamic assessments [44]. Ibrexafungerp exhibited no clinically relevant effects on heart rate or PR and QRS intervals. No clinically meaningful effect of ibrexafungerp on the QTcF interval was observed at plasma concentrations up to 4000 ng/mL after IV administration in healthy subjects.

Results from in vitro studies indicated that ibrexafungerp is a substrate of CYP3A4 and a potential inhibitor of cytochrome (CYP) 2C8 [42,45]. In healthy subjects, the interaction potentials of ketoconazole and diltiazem (CYP3A4 inhibitors), rosiglitazone (CYP2C8 substrate), and tacrolimus (CYP3A4 substrate) were evaluated after single or multiple doses co-administered with ibrexafungerp [46]. No clinically relevant effects of ibrexafungerp on CYP2C8 inhibition or CYP3A4 substrates were observed, although a dosage adjustment for ibrexafungerp may be needed when co-administering with potent CYP3A4 inhibitors. Thus, ibrexafungerp shows low potential for CYP-mediated drug interactions at therapeutic exposures.

### 2.4. Clinical Development

Nineteen Phase 1, three Phase 2, and two Phase 3 clinical studies were completed with ibrexafungerp, the latter two (VANISH-303 and VANISH-306), in women with vulvovaginal candidiasis (VVC) (https://clinicaltrials.gov: NCT03734991 and NCT03987620). In both Phase 3 studies, the rate of clinical cure (complete resolution of all vaginal signs and symptoms at Day 10) and mycological eradication were significantly greater with ibrexafungerp than placebo. The most common adverse events reported with ibrexafungerp were mild gastrointestinal disturbances when administered to >1200 patients and healthy subjects.

Currently, ibrexafungerp is undergoing further clinical development in ongoing studies, including CANDLE-304 (clinicaltrials.gov: NCT04029116), a Phase 3 study in women with recurrent VVC, SCYNERGIA, a Phase 2 study of ibrexafungerp combined with voriconazole in patients with invasive aspergillosis (clinicaltrials.gov: NCT03672292), FURI, an open-label study in patients with refractory invasive fungal infections (clinicaltrials.gov: NCT02244606), and CARES (clinicaltrials.gov: NCT03363841), an open-label, emergency protocol study of patients with invasive infections due to *C. auris*.

## 3. Ibrexafungerp for *Candida auris*

### 3.1. In Vitro Activity

The in vitro activity of ibrexafungerp was tested against 16 *C. auris* clinical isolates obtained from Germany, Japan, India, and South Korea [27]. The MIC_90_ for ibrexafungerp was 1 μg/mL. Fluconazole and amphotericin B exhibited less in vitro activity against *C. auris* with MIC_90_ values of >64 and 4 μg/mL, respectively, while the MIC_90_ values for anidulafungin, caspofungin, and micafungin were 0.25 μg/mL, 1 μg/mL, and 1 μg/mL, respectively.

The in vitro activity of ibrexafungerp was evaluated against a global collection of 100 isolates of *C. auris* representing each of the four clades of *C. auris* known at that time [24]. MICs for ibrexafungerp ranged from 0.0625 to 2 μg/mL, with an MIC_50_ of 0.5 μg/mL and MIC_90_ 1 μg/mL. MIC values for anidulafungin, caspofungin, and micafungin ranged from 0.03 up to >16 μg/mL. Among seven *C. auris* isolates exhibiting elevated MIC values for echinocandins, the ibrexafungerp MIC ranged from 0.5 to 1.0 μg/mL.

Ibrexafungerp and six comparator antifungal agents were evaluated against 122 *C. auris* isolates [33]. The MIC range for ibrexafungerp was 0.06 to 2.0 μg/mL. A wide distribution of MIC values was reported for anidulafungin and micafungin, ranging from 0.016 to >32 and 0.03 to >32 μg/mL, respectively (Table 1). All but one *C. auris* isolate were resistant to fluconazole. Out of 122 isolates, 8 displayed high MIC values for echinocandins associated with *fks* mutations (S639F Fks1 alteration). The MIC for ibrexafungerp for these eight resistant isolates ranged from 0.25 to 0.5 μg/mL.

Among 102 *C. auris* isolates with variable resistance to amphotericin B, flucytosine, azoles, and echinocandins, the ibrexafungerp MIC_50_ for 97 isolates ranged from 0.06–0.5 μg/mL, and the median and mode MIC were both 0.5 μg/mL [32]. Ibrexafungerp also showed activity against five *C. auris* isolates considered to be pan-resistant, with a low MIC_50_ range of 0.12 to 1 μg/mL.

Data were compiled from four studies reporting the in vitro activity of ibrexafungerp against 445 *C. auris* clinical isolates [47]. Most isolates were obtained from the United States and India and included 32 isolates with increased MIC values to echinocandins. The MIC_50_ and MIC_90_ for ibrexafungerp across all isolates tested were 0.5 μg/mL and 1.0 μg/mL, respectively (Table 2). Among 32 *C. auris* isolates with echinocandin resistance, MIC values for ibrexafungerp ranged from 0.5 μg/mL to 1.0 μg/mL. One isolate displayed high MIC values for echinocandins and showed reduced sensitivity (>2 dilutions vs. the mode) to ibrexafungerp, and this isolate exhibited elevated MIC values to anidulafungin, caspofungin, and micafungin (MIC = 1 μg/mL), luconazole (MIC > 256 μg/mL), and amphotericin B (MIC = 1 μg/mL). Thus, ibrexafungerp exhibits in vitro activity against a broad collection of *C. auris* isolates, including most echinocandin-resistant isolates.

The ability of *Candida* species to form biofilms is associated with catheter and device-related infections and may play a role in *C. auris* infections considering that many affected individuals are in intensive care units with intravascular lines. In this regard, 97% of patients infected with *C. auris* had central venous catheters (Sayeed et al., 2019) [48], and a retrospective analysis demonstrated significantly higher use of central venous catheters in patients infected with this multidrug- resistant *Candida* [49]. The activity of ibrexafungerp against *C.* *auris* biofilms was evaluated [27]. Following 48 h of incubation, metabolic activities of biofilms were measured. Images and thicknesses of biofilms growing in the presence or absence of a drug were captured using confocal scanning laser microscopy. Quantitation of the metabolic activity of *C. auris* biofilms was performed using a biochemical assay, the 2,3-bis(2-methoxy-4-nitro-5-sulfophenyl)-5-[(phenylamino) carbonyl]-2H-tetrazolium hydroxide (XTT) reduction assay, as described previously [27,50]. Ibrexafungerp demonstrated activity against *C. auris* biofilms by reducing biofilm thickness and metabolic activity.

**Figure 3:**
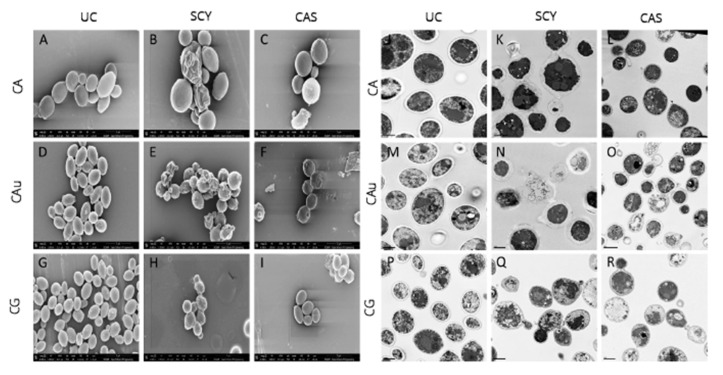
SEM of CA UC (**A**), SCY treated CA (**B**), CAS treated CA (**C**), CAu UC (**D**), SCY treated CAu (**E**), CAS treated CAu (**F**), CG UC (**G**), SCY treated CG (**H**), CAS treated CG (**I**), and TEM of CA UC (**J**), SCY treated CA (**K**), CAS treated CA (**L**), CAu UC (**M**), SCY treated CAu (**N**), CAS treated CAu (**O**), CG UC (**P**), SCY treated CG (**Q**), and CAS treated CG (**R**). (Hager et al., 2018) [51].

The effects of ibrexafungerp and caspofungin on the morphology of *C. albicans*, *C. auris*, and *C. glabrata* were studied using scanning and transmission electron microscopy [51]. When evaluated at respective MIC_50_ levels, ibrexafungerp exhibited a profound effect on cellular morphology in caspofungin-resistant organisms, possibly indicative of a difference in target engagement between ibrexafungerp and echinocandins (Figure 3). Untreated control *C. auris* cells showed well-defined, oval-shaped yeast morphology, as well as several budding yeasts. In contrast, cells exposed to ibrexafungerp (at a concentration of 1 µL MIC) exhibited a severely distorted yeast cell topography, including cell collapse, deformed cellular appearance, irregular budding, and cells that were fused together and unable to undergo cell division [27].

### 3.2. In Vivo Activity

The in vivo efficacy of ibrexafungerp for *C. auris* was evaluated in a disseminated murine mouse model [37]. Immunocompromised mice were randomized to ibrexafungerp 10, 20, or 30 mg/kg twice daily (BID) vs. a vehicle given by oral gavage. At Day 7, the fungal burden in kidney tissue was reduced by all doses of ibrexafungerp, with a significant difference for the 30 mg/kg dose vs. vehicle. At Day 14, survival rates were 60–70% with ibrexafungerp vs. 20% with vehicle control. Exposures in mice dosed with ibrexafungerp 10, 20, or 30 mg/kg BID were consistent with steady-state plasma exposure (AUC_0–24_) of 8.4, 24.3, and 40.2 ug*h/mL, respectively. These results demonstrate potent antifungal activity of ibrexafungerp against *C. auris*.

*C. auris* colonization is a major problem in hospitals and long-term care facilities. In order to understand the ability of ibrexafungerp to potentially decolonize the skin of *C. auris*, a study was performed looking at the in vivo efficacy of ibrexafungerp in a cutaneous infection model in Guinea pigs [36]. Animals were treated with ibrexafungerp 10, 20, or 30 mg/kg BID by oral gavage, micafungin 5 mg/kg once daily IP, or vehicle by oral gavage, and prednisone 30 mg/kg SC was given one day before and three days after infection. Tissue burden at Day 7 was lower with all active treatments vs. vehicle. Animals dosed with ibrexafungerp 10, 20, or 30 mg/kg BID showed systemic exposures (AUC_0–24_) of 2.8, 5.6, and 15 ug*h/mL. Examination of Periodic Acid-Schiff (PAS)-stained skin sections revealed that sections obtained from untreated control animals showed yeast cells, demonstrating that the skin was infected with *C. auris*. In contrast, examination of multiple skin sections obtained from animals treated with either ibrexafungerp or micafungin did not reveal yeast cells at any of the dose levels tested, indicating that the *C. auris* infection was cleared. There was no significant difference in clinical scores between the treatment groups [36]. Thus, no fungal elements were observed with ibrexafungerp or micafungin from histological examination.

### 3.3. Clinical Experience

CARES is an open-label study of oral ibrexafungerp in patients with documented candidiasis or candidemia due to *C. auris* who were treatment naïve or refractory to or intolerant of standard-of-care antifungal agents (clinicaltrials.gov: NCT03363841). Patients were treated with oral ibrexafungerp 750 mg twice daily for two days, then 750 mg once daily for up to 90 days.

In the first two patients from CARES with candidemia due to *C. auris*, a complete response after 17 and 22 days of treatment was reported with ibrexafungerp [52]. The first patient was a 58-year-old male admitted to the ICU with pneumonia and septic shock. Antibiotics were given together with empiric IV fluconazole. When *C. auris* was isolated from blood cultures, antifungal therapy was switched to IV micafungin. However, blood cultures remained positive for *C. auris* after five days, and the patient was switched to ibrexafungerp for 17 days. Subsequent blood cultures at Day 3 of ibrexafungerp therapy were negative for *C. auris*, and the patient was considered to have a complete response at the end of therapy. Ibrexafungerp-related adverse events were mild loose stools from days two through four of therapy.

The second patient was a 64-year-old female admitted to the hospital with pneumonia, fever, and hypotension. When *C. auris* was isolated from blood cultures, ibrexafungerp was initiated. A blood culture collected on Day 3 of ibrexafungerp therapy remained positive for *C. auris* and subsequent cultures at Days 9 and 21 were reported negative. The patient improved clinically, received ibrexafungerp for 22 days and was considered a complete response at the end of therapy. No ibrexafungerp-related adverse events were reported.

### 3.4. Echinocandin Resistance and C. auris

For echinocandins, the primary mechanism of resistance in *C. auris* species comprises the *fks*1 and *fks*2 genes, where mutations of the S639F, S639P, and S639Y amino acid sequences were identified as the cause of elevated MICs to echinocandins [9]. Among 350 *C. auris* isolates from India, 2% were echinocandin-resistant due to the *fks*1 mutation expressing the S639F sequence [53]. A similar finding was reported from Kuwait, where 3 (1.0%) of 314 *C. auris* isolates were echinocandin-resistant due to the *fks*1 mutation expressing the S639F sequence [54]. Four additional *C. auris* isolates from a total of 106 isolates were resistant to all tested echinocandins (MIC ≥ 4 μg/mL) and contained an S639F mutation in *fks*1 [55].

Biagi et al. [56] reported a patient with recurrent candidemia due to *C. auris* that was echinocandin-resistant but azole-sensitive, who expressed the *fks*1 mutation for the S639P sequence. A single *C. auris* isolate was identified in the UK that displayed 5-flucytosine and echinocandin resistance; echinocandin resistance was due to *fks*1 mutation for the S639Y sequence [57].

Among *C. auris* isolates from India, 8 of 122 with the S639F sequence were echinocandin-resistant with MICs of 4–32 μg/mL; the ibrexafungerp MIC values for these same isolates ranged from 0.25–0.5 μg/mL [33].

Ostrowski and colleagues [18] described three cases of *C. auris* among 801 patients in New York state with confirmed *C. auris* that were pan-resistant, i.e., resistant to fluconazole, amphotericin B, and echinocandins. All three patients with pan-resistance were on mechanical ventilators; two died within two to four weeks from isolation of a pan-resistant *C. auris*, and the third died at 10 months. MICs for fluconazole were >256 μg/mL, 2 μg/mL for amphotericin B, and 2–16 μg/mL for echinocandins. From the same laboratory, an analysis of the susceptibility of five pan-resistant *C. auris* isolates, defined as in vitro resistance to more than two azoles, all echinocandins, and amphotericin B, reported MIC values to fluconazole (>256 μg/mL), amphotericin B (2 μg/mL), and echinocandins (ranging from 2 to >16 μg/mL) [15]. However, all these pan-resistant isolates exhibited MICs for ibrexafungerp ranging from 0.12 to 1 μg/mL, which were within the wild-type MIC range reported for *C. auris*.

## 4. Summary and Conclusions

In the past decade, *C. auris* has emerged as a critical public health concern because of its persistence in the environment, contagious nature, and high morbidity and mortality. Unfortunately, rates of resistance to antifungal drugs among *C. auris* isolates continue to increase with almost universal resistance to fluconazole and growing resistance to other azoles and antifungals, including echinocandins. New antifungal agents in clinical development may provide more effective treatment options to address the growing impact of *C. auris*. Ibrexafungerp offers the advantages of oral administration, a favorable PK profile, a well-characterized safety/tolerability profile in >1200 patients/subjects, a low risk for cross-resistance to echinocandins, and consistently potent in vitro activity against *C. auris*, including echinocandin-resistant isolates. Animal studies looking at the tissue distribution of ibrexafungerp achieved high concentrations in the skin, an attribute that may be of importance to limit *C. auris* skin colonization, with a potential impact of limiting transmission. Ibrexafungerp demonstrates activity across a range of invasive fungal diseases as a monotherapy but also in combination [58,59]. Early clinical evidence from an emergency-use Phase 3 study of ibrexafungerp for invasive candidiasis due to *C. auris* is promising. Additional preclinical and clinical evidence are awaited to confirm the role of ibrexafungerp in treating infections due to *C. auris,* as well as other causes of invasive fungal disease.

## Figures and Tables

**Table 1:** In vitro activity of ibrexafungerp and comparators against *C. auris* isolates [33].

Drug (No. of Isolates)	MIC_50_^a^	Modal MIC	MIC Range
Ibrexafungerp (*n* = 122)	0.5	0.5	0.06–2
Anidulafungin	0.125	0.06	0.016–>32
Micafungin	0.125	0.125	0.03–>32
Amphotericin B	1	1	0.5–1
Fluconazole	≥64	≥64	0.5–≥64
Voriconazole	0.5	Bimodal	≤0.004–4
Isavuconazole	0.125	Trimodal	≤0.004–2

^a^ μg/mL; Ibrexafungerp minimum inhibitory concentration (MIC) values for eight isolates with S639F *fks1* mutations ranged from 0.25 to 0.5 μg/mL.

**Table 2:** In vitro activity of ibrexafungerp against a compilation of 445 *C. auris* isolates [47].

Reference	No. of Isolates	MIC, μg/mL			
		MIC_50_	MIC_90_	Mode	MIC Range
Berkow et al., 2017 [24]	107	1	1	1	0.0625–2
Larkin et al., 2017 [27]	16	1	1	1	0.5–1
Zhu et al., 2020 [32]	200	0.5	1	0.5	0.0625–8
Arendrup et al., 2020 [33]	122	0.5	1	0.5	0.0625–2
Overall	445	0.5	1	0.5	0.625–8
